# P-1507. *In Vitro* Activity of Ceftazidime-avibactam against Enterobacterales Isolates Producing Multiple β-lactamases Collected Globally as a Part of the ATLAS Global Surveillance Program from 2018-2022

**DOI:** 10.1093/ofid/ofae631.1676

**Published:** 2025-01-29

**Authors:** Mark Estabrook, Henry Li, Gregory Stone, Katherine Perez, Daniel F Sahm

**Affiliations:** IHMA, Schaumburg, Illinois; IHMA, Schaumburg, Illinois; Pfizer, Inc., Groton, Connecticut; Pfizer, Inc., Groton, Connecticut; IHMA, Schaumburg, Illinois

## Abstract

**Background:**

Ceftazidime-avibactam (CAZ-AVI) is a β-lactam/β-lactamase inhibitor combination approved to treat infections caused by Gram-negative organisms. Notably, CAZ-AVI has activity against Enterobacterales producing Class A, C, and D β-lactamases, but not Class B metallo-β-lactamases (MBLs). The *in vitro* activity of CAZ-AVI and comparator agents against clinical Enterobacterales isolates producing one or more β-lactamase collected as a part of the ATLAS global surveillance program (2018-2022) was evaluated.

**Figure:**
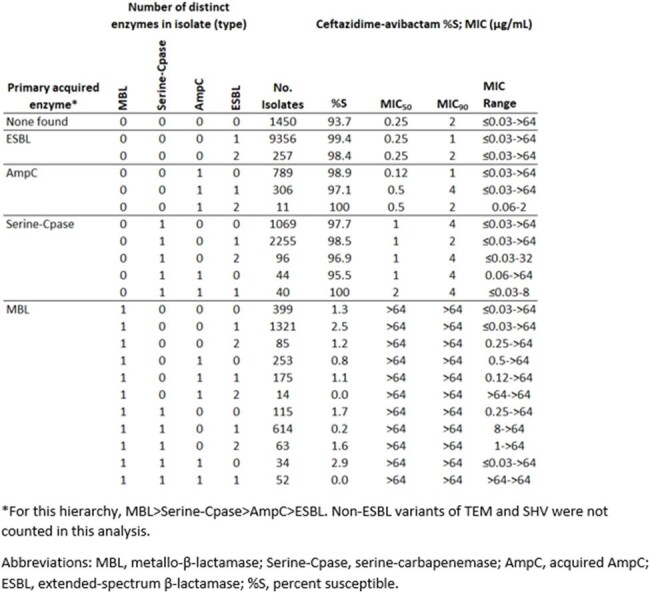

**Methods:**

89,316 isolates from 228 medical centers in 57 countries (excluding mainland China, Canada, and the USA) were collected and tested for susceptibility using the broth microdilution method according to CLSI guidelines. Analysis was performed with CLSI 2024 breakpoints. Isolates testing with meropenem MIC values >1 µg/mL or *Escherichia coli, Klebsiella pneumoniae, K. oxytoca,* or *Proteus mirabilis* isolates testing with ceftazidime and/or aztreonam MIC values >2 µg/mL were screened for β-lactamase genes by PCR, which were sequenced when identified.

**Results:**

One or more extended-spectrum β-lactamase (ESBL), acquired AmpC, serine-carbapenemase, or MBL was identified among 17,348/18,798 isolates characterized. Against isolates producing ESBLs (51.1%), susceptibility to CAZ-AVI was similar if the isolate carried one ESBL (99.4%) or two ESBLs (98.4%). Against acquired AmpC-producing isolates (5.9%), CAZ-AVI susceptibility was similar if the isolates co-carried 0, 1, or 2 ESBLs (98.9%, 97.1%, and 100% susceptible, respectively). Against isolates carrying serine-carbapenemases (18.6%), CAZ-AVI activity was similar regardless of co-carriage of acquired AmpC and/or one or more ESBLs (95.5-100% susceptible). CAZ-AVI was not active against isolates that carried an MBL (16.6%), regardless of other enzyme carriage (0-2.9% susceptible).

**Conclusion:**

These results highlight that the number of distinct β-lactamases have little association with susceptibility to CAZ-AVI, while the type of β-lactamase (MBL or non-MBL) has a greater association.

**Disclosures:**

**Mark Estabrook, MS**, Pfizer, Inc.: Advisor/Consultant **Henry Li, MS in Biotechnology**, Pfizer, Inc.: Advisor/Consultant **Daniel F. Sahm, PhD**, Pfizer, Inc.: Advisor/Consultant

